# Microbial Protein as a novel ingredient in pet food: A food preference study in dogs and cats

**DOI:** 10.1371/journal.pone.0356767

**Published:** 2026-09-23

**Authors:** Ally Motta de Matos, A.L.B. Cruz

**Affiliations:** MicroHarvest Unipessoal Lda., Lisbon, Portugal; Lusofona University of Humanities and Technologies: Universidade Lusofona de Humanidades e Tecnologias, PORTUGAL

## Abstract

The increasing demand for protein sources in pet nutrition has driven growing interest in microbial proteins (MP) as viable alternatives to traditional animal proteins. However, their successful incorporation into pet food formulations depends not only on nutritional adequacy but also on short-term food preference. This study evaluated the palatability of dry extruded diets containing 10% microbial protein in dogs and cats using an in-home two-bowl preference test and complemented these findings with independent laboratory characterization to support interpretation of the observed preferences. Palatability was assessed in 30 dogs and 30 cats over four meals by comparing MP-containing diets with macronutrient matching control diets differing only in protein source. Trials were conducted in-home to ensure natural feeding behavior, and data on food consumption, first approach, and primary consumption were collected. In both species, animals showed a statistically significant preference for diets containing microbial protein. Dogs consumed 72% of the MP-containing diet compared to 50% of the control (Wilcoxon signed-rank test, *p* = 0.002), whereas cats exhibited a consumed 51% of the MP-containing diet compared with 34% of the control diet (*p* = 0.001). Behavioral observations indicated that both dogs and cats more frequently approached and consumed the MP-containing diets first, a preference for the MP-containing diets. To contextualize these palatability outcomes, control and MP-containing diets were analyzed by an independent laboratory for proximate composition, amino acid and fatty acid profiles, selected minerals, and targeted volatile compounds. The analysis confirmed comparable macronutrient composition between diets, with MP-containing diets showing a complete amino acid profile, higher tryptophan content, and a lower omega-6: omega-3 ratio. Targeted volatile screening did not reveal analytical indicators typically associated with off-flavors in either of the diets. Together, these findings indicate that the preference observed in dogs and cats for MP-containing diets occurred within a nutritionally comparable formulation framework and may be supported by favorable analytical characteristics relevant to short-term food preference. This exploratory study provides complementary evidence supporting the feasibility of microbial protein inclusion in extruded pet food formulations and lays the groundwork for future research focused on long-term feeding outcomes, health and optimization of palatability. The present findings are limited to short-term food preference under the conditions evaluated and should not be interpreted as evidence of long-term acceptance or health effects.

## Introduction

The increasing trend of pet humanization has shifted the focus towards providing optimal nutrition to companion animals, which are now often regarded as family members [1]. This shift is accompanied by rising concerns about the availability and environmental impact of traditional protein sources used in pet food. Global meat demand is projected to increase by 75% by 2050 due to population growth [2]. Notably, this projection does not account for the concurrent increase in the global pet population, which further amplifies the demand for protein-rich diets to meet animals’ nutritional requirements. Insufficient intake of essential amino acids may lead to nutritional deficiencies and associated health complications [3].

As global protein demand continues to rise amidst stagnant production volumes of traditional sources, alternative proteins with lower environmental footprints have gained attention as viable solutions [4]. Microbial proteins have emerged as promising alternatives to traditional animal proteins in both human and animal nutrition [5]. Produced through efficient and scalable fermentation processes using microorganisms like fungi, yeast, and bacteria, these proteins offer a unique combination of nutritional and environmental benefits. Prior studies have demonstrated that Microbial proteins exhibit high digestibility and an excellent amino acid profile, making them nutritionally comparable or even superior to conventional protein sources [6,7]. These characteristics are particularly advantageous for meeting the dietary requirements of companion animals, which depend on balanced amino acid intake for optimal health. Additionally, Microbial proteins production requires significantly less agricultural land and water while emitting fewer greenhouse gases compared to conventional protein sources, making them an attractive solution for addressing the challenges of sustainable protein production [5].

In the context of pet food, however, the success of such alternatives depends on more than just sustainability and availability. A viable protein source must meet nutritional standards while also being palatable to pets [4]. This integration of environmental, nutritional, and sensory criteria is critical for ensuring the market success of alternative proteins in pet food formulations. The acceptance of alternative proteins is heavily influenced by sensory characteristics such as taste, texture, and aroma [4]. As a result, palatability is a critical determinant in ensuring that pets readily accept these novel protein sources. Consequently, palatability trials are indispensable for evaluating how pets perceive these proteins within their diets and for facilitating their seamless incorporation into pet foods without compromising sensory appeal or overall palatability [8]. Evaluating the palatability of pet foods typically involves standardized “consumption” tests conducted in controlled environments like kennels or in a natural home setting via in-home use tests (IHUT). These tests offer flexibility in terms of the breeds and sizes of animals used, allowing researchers to gather data across diverse pet populations [4,8]. Common methods include single-bowl acceptance tests and two-pan preference tests, which assess food appeal and preferences. Acceptability is defined as the extent to which an animal consumes sufficient calories from a food item to maintain health and performance, independent of sensory characteristics such as taste or smell [4,8]. Preference, by contrast, reflects a comparative choice for one food over another, influenced by sensory factors like aroma and texture [9]. Aroma plays a particularly significant role, as dogs and cats rely heavily on their olfactory senses, enhanced by the vomeronasal organ (Jacobson’ s organ), which amplifies scent perception and is influenced by environmental factors like moisture and temperature [4] The “first-choice” response and animal’s initial approach to and consumption of a food is often used to assess the impact of aroma. Occasionally, animals reject foods accepted by others due to “conditioned aversion” resulting from prior negative experiences [10]. Behavioral responses like “neophobia” (fear of new foods) and “neophilia” (preference for novel foods) are frequently observed, particularly in cats but also in dogs [4]. Developing alternative protein sources for pet food requires balancing sustainability, nutrition, and palatability. Addressing these factors is essential to reduce the environmental impact of traditional meat production while ensuring that pet food remains both desirable and nutritious for companion animals [3,4,8].

The objective of this study was to evaluate the short-term preference for dry pet food containing microbial protein using a two-bowl in-home use test (IHUT) in dogs and cats. This study investigates the impact of replacing traditional animal protein sources with MP on the palatability of dry extruded diets for dogs and cats. By applying established palatability testing methods and evaluating behavioral responses to novel foods, we aim to determine whether this substitution decreases, maintains, or enhances the food preference. In addition, independent laboratory characterization of experimental diets was conducted to support interpretation of the observed palatability outcomes within a nutritionally comparable formulation framework. This work builds on previous findings regarding the high digestibility and balanced amino acid profile of microbial proteins, while emphasizing their potential contribution to more sustainable pet nutrition. Our central hypothesis is that microbial protein can be incorporated into pet food formulations without compromising palatability and may even increase the food preference in dogs and cats.

## Materials and Methods

### Study design

For the palatability trial, In-Home Use Testing (IHUT) was used to allow for more representative outcomes of pet preferences [11]. The study was designed according to the standardized IHUT procedures routinely used by the independent contract research organization (Sense Test, Portugal), reflecting established industry practice for companion animal palatability assessments. Thirty animals per species were enrolled, consistent with the sample size commonly used in commercial in-home use test (IHUT) palatability studies. All animals were registered in a database that included detailed characteristics such as breed and body weight. Dogs ranged from 3 to 16 years of age, had a body weight between 3 and 10 kg (mean 6 kg), and represented 11 breeds. Cats ranged from 3 to 17 years of age, weighed between 2 and 10 kg (mean 5 kg) and represented 6 breeds.

All animals were familiarized with the type of food being tested, specifically commercially available industrially produced dry food, as commonly applied in palatability trials conducted under IHUT conditions [11]. According to owner reports, all animals were healthy and had no history of chronic diseases or food allergies.

During the trial, each animal received the same amount of food they were accustomed to consuming in their daily routine. Feeding amounts were adjusted based on age and body weight to ensure consistency with normal dietary intake patterns. This approach aimed to minimize variability related to feeding habits while maintaining the nutritional requirements of the animals, in line with standard IHUT methodology [11].

### Control and test formulas

The same two dry extruded diets were evaluated in the palatability trial: a control diet and a test diet containing MP. The test diet was formulated by including microbial protein at a level of 10% (w/w), hereafter referred to as the *MP-containing diet*. The control and MP-containing diets were formulated to achieve comparable nutritional targets and differed primarily in their protein source. Minor analytical differences observed after manufacturing reflect normal formulation and analytical variability.

The control diet was formulated to mimic a commercially available complete and balanced formula marketed with a fish and rice flavor profile. Its composition included cereals (12% rice), meat and animal derivatives (34%), poultry fat, krill (3%), beet pulp (1.6%), hydrolyzed animal protein, yeast, carob, minerals, vitamins, and *Yucca schidigera* (0.001%).

The MP-containing diet was identical to the control diet in overall nutritional composition but differed in ingredient profile by the inclusion of MP, which fully replaced fish meal and partially replaced poultry meal. Due to the inherently low-fat content of microbial protein, poultry fat was adjusted to maintain comparable nutritional targets between diets. The composition of both diets is presented in Table 1, and their analytical constituents determined after manufacturing are reported in Table 2.

**Table 1:** Ingredient composition of experimental pet food formulations used in palatability assessment.

Ingredient composition (%)		
	Control Formula (Fish and Rice)	Test Formula (containing MP)
Cereals	51%	51%
Poultry Meal	28%	22%
MP	–	10%
Fish Meal	3.5%	–
Meat and bone Meal	2%	2%
Poultry fat	7%	7.5%
Krill	3%	3%
Beet pulp	2%	2%
Hydrolyzed animal protein	2%	2%
Carob	0.2%	0.2%
Yeast	0.3%	0.3%
Premix	0.5%	0.5%
Yucca schidigera	0.001%	0.001%

The same formulations were used for both the dog and cat palatability studies.

**Table 2:** Analytical composition of experimental diets and estimated metabolizable energy.

	Analytical Composition	
	Control Formula (Fish and Rice)	MP-containing diet
Crude Protein	30%	30%
Crude Fat	13%	13%
Crude Fiber	2.5%	2.5%
Raw Ash	8.5%	9%
Moisture	8%	8%
Estimated metabolized energy (kcal/kg)	3,485	3,468

Estimated metabolizable energy (ME) was calculated using the modified Atwater factors (3.5 kcal/g crude protein, 8.5 kcal/g crude fat, and 3.5 kcal/g nitrogen-free extract).

Both experimental diets were manufactured under controlled and identical conditions at Ovargado S.A. (São João de Ovar, Portugal) using an industrial-scale single-screw extrusion system (Andritz), with a minimum batch capacity of approximately 3,000 kg. The base mixture was preconditioned with direct steam injection and water addition to facilitate starch gelatinization and initial thermal processing. Extrusion was performed under controlled thermal and mechanical parameters, with barrel temperatures ranging from 120 °C to 140 °C. The extrudate was cut at the die face to form uniform kibbles, which were subsequently dried to a final moisture content below 10%.

Post-extrusion, the dried kibbles were coated using a rotary drum coater operated at ambient temperature and atmospheric pressure. A portion of the total poultry fat (included at 7.0–7.5% of the formulation) was first applied, followed by powdered poultry digest (0.2%, w/w) as a palatability enhancer. The same post-extrusion coating procedure, including identical poultry fat and poultry digest application rates, was used for both the control and MP-containing diets.

### Testing locations and product delivery

The experimental trials were conducted in the animals’ habitual feeding environments, predominantly in their domestic settings (Portuguese households). Test products were delivered to participants by Sense Test, Lda. (Vila Nova de Gaia, Portugal) technicians or representatives. During individual meetings, the trial conditions, protocols, and requirements were explained, and participants were provided with written instructions, which they were required to acknowledge before beginning the trial. Pet owners were instructed to feed the diets as part of their pets’ routine feeding practices to ensure consistency. The products were delivered in appropriate containers, each clearly labeled with a code and a generic description of the sample. All samples were coded to maintain blinding and minimize bias throughout the trial. The use of coded labeling ensured that participants remained unaware of the specific formulation of the diets during the study, thereby mitigating any potential subjective influence on feeding behavior and trial outcomes.

### Two-bowl preference test

The two-bowl preference test was used to assess short-term food preference and was carried out as follows: each animal was simultaneously presented with two bowls of food, one containing the MP-containing diet and the other containing the control diet [4,8]. The test was conducted over two consecutive days (four meals per animal), representing a short-term assessment of food preference. To minimize potential bias, the positions of the bowls were alternated during each of the four meals. Both bowls were placed in the animals’ feeding area with food portions standardized to identical amounts for both samples, corresponding to the quantities typically provided to the animals in their daily meals. Owners were instructed not to provide treats, table scraps, or any other foods during the two-day study period and to use the feeding bowls provided by Sense Test. The trial was conducted under routine home-feeding conditions to minimize disruption of the animals’ usual feeding behavior.

### Data collection and analysis

For each meal, owners used digital scales to record the weight of the empty bowl, the bowl containing food before feeding, and the bowl with the remaining food after feeding. Food consumption was calculated as the difference between the amount of food offered and the amount remaining after each meal. Food consumption data from the four meals were aggregated for each animal prior to statistical analysis, and each animal was considered the experimental unit. Owners also recorded the first bowl approached, the first bowl from which the animal consumed food, and whether the total food intake during the test period was higher, lower, or similar to the animal’s usual intake. These observations were summarized descriptively. Statistical analyses were performed using XLSTAT (Lumivero, USA) and Microsoft Excel. Differences in food intake between the MP-containing and control diets were assessed using the Wilcoxon signed-rank test. Statistical significance was set at *p* < 0.05. First approach and first consumed were reported descriptively as percentages.

### Analytical characterization of experimental diets

Independent laboratory analyses were conducted to characterize control and MP-containing diets and to support interpretation of the palatability results. All analyses were performed by Eurofins Food Testing Lisboa (Lisbon, Portugal) using validated internal methods or internationally recognized standard methods, as specified in the analytical reports.

Proximate composition, including crude fat (after acid hydrolysis) and crude ash, was determined using internal gravimetric methods. Fatty acid profiles were analyzed by gas chromatography with flame ionization detection (GC-FID), with results expressed both as relative percentages and as absolute values (g/100 g) based on fat content.

Amino acid composition was determined after acid hydrolysis according to ISO 13903:2005 using ion chromatography with UV detection (IC-UV). Sulfur-containing amino acids (cysteine and cystine) and methionine were analyzed following oxidative hydrolysis, while tryptophan was quantified separately using liquid chromatography with fluorescence detection (LC-FLD) according to EU Regulation 152/2009.

Selected minerals were quantified using inductively coupled plasma optical emission spectrometry (ICP-OES) following an internal validated method (CON-PV 00006, 2023−12).

To support interpretation of palatability outcomes, a targeted screening of aroma-related volatile compounds was performed using gas chromatography–mass spectrometry (GC-MS) according to an internal laboratory method.

## Results

### Canine Diets

An IHUT trial was conducted with 30 dogs from different breeds (average body weight 5.9 ± 2.4 kg) to assess short-term food preference of formulations with or without microbial protein. Both diets were formulated to achieve comparable nutritional targets and differed primarily in their protein source. Overall food intake remained similar to the animals’ usual intake, with 91% ± 3% of participants reporting that their dogs consumed the usual amount or more than their regular meals. Only four participants (13%) reported minor alterations in fecal consistency, with two reporting firmer stools and two reporting softer stools.

**Fig 1:**
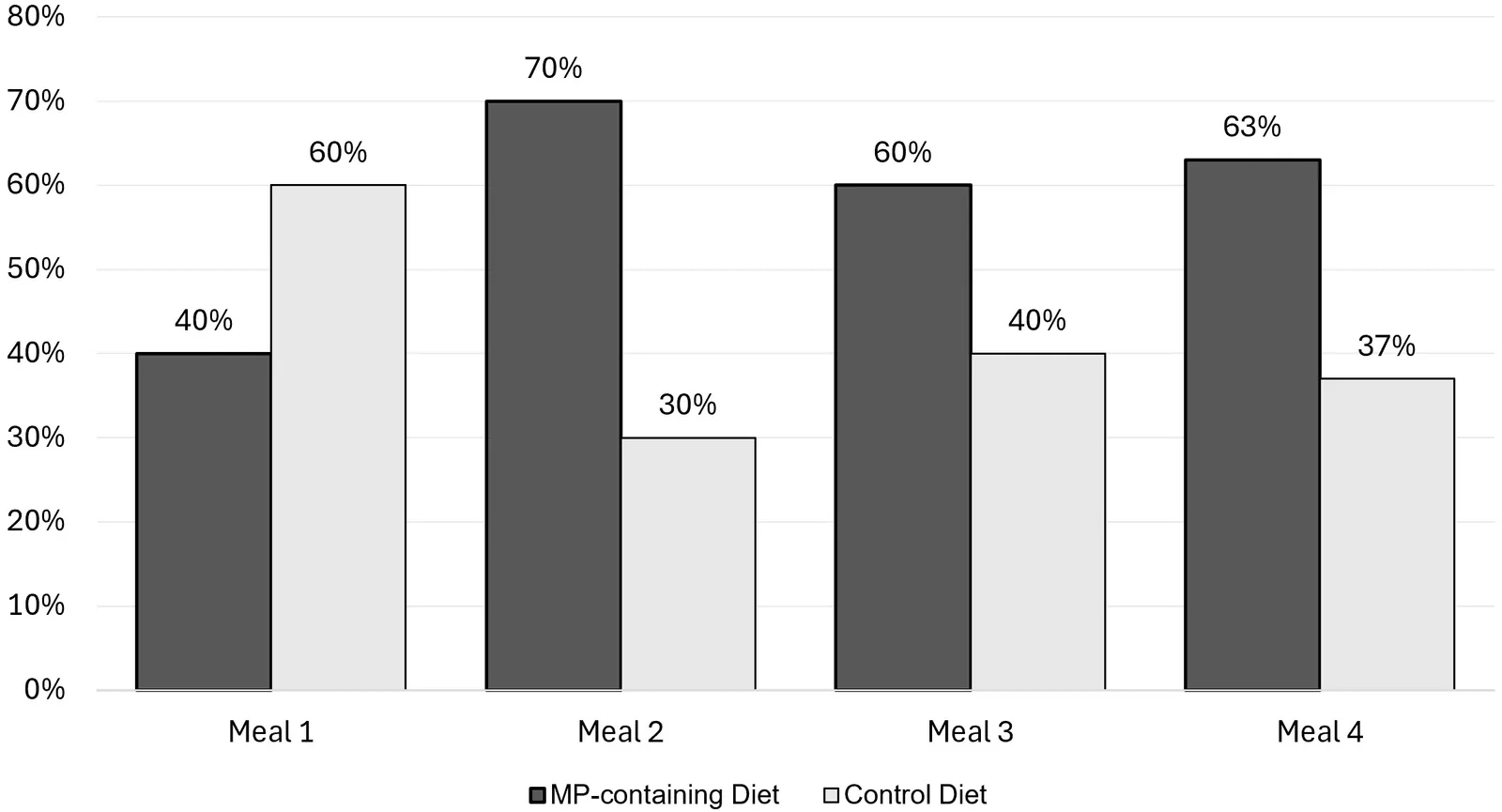
First approach (Dogs). Percentage of observations in which dogs first approached the MP-containing diet or the control diet during each meal of the two-bowl preference test. Data are presented descriptively as percentages.

**Fig 2:**
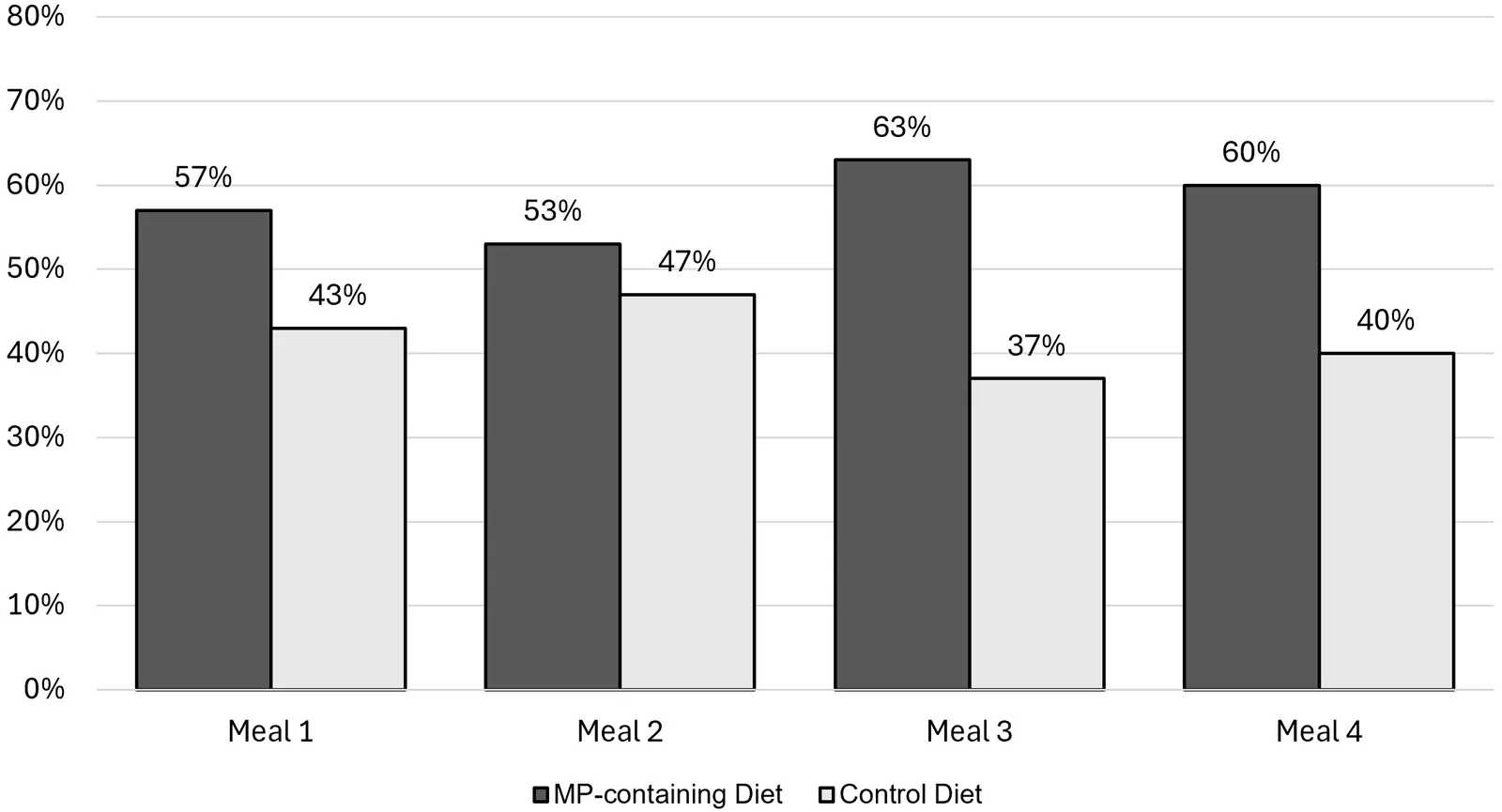
Primary consumption (Dogs). Percentage of observations in which dogs first consumed the MP-containing diet or the control diet during each meal of the two-bowl preference test. Data are presented descriptively as percentages.

**Fig 3:**
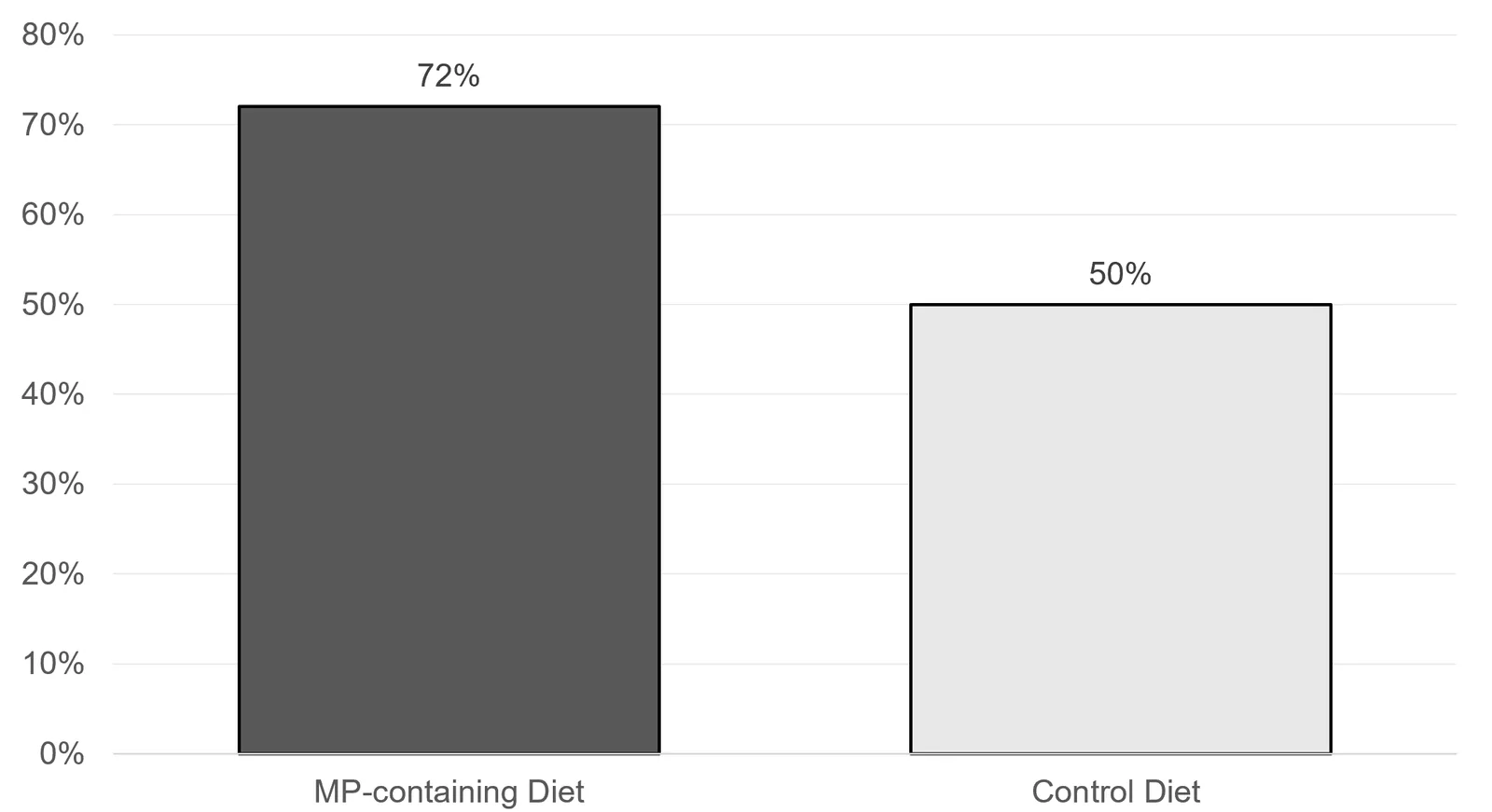
Food intake (Dogs). Mean proportion of the MP-containing diet and control diet consumed by dogs during the two-bowl preference test. Bars represent the average percentage of total food intake across the study. Food intake was compared using the Wilcoxon signed-rank test (*p* = 0.002).

Regarding food preference, the two-bowl preference test revealed that dogs exhibited a statistically significant preference for the MP-containing diet compared with the control diet. Across the four meals, 58% of dogs first approached the MP-containing diet and 58% consumed it first, compared with 42% for the control diet (Figs 1 and 2). Fig 3 shows that dogs consumed a significantly greater proportion of the MP-containing diet than the control diet (72% vs. 50%; Wilcoxon signed-rank test, p = 0.002).

### Feline Diets

A similar IHUT trial was performed, but this time with 30 cats (average weight 5.3 ± 2.1 kg). Three out of four cats (75% ± 2%) were reported to have maintained or increased their food consumption during the trial compared to their regular meals. Overall, a statistically significant difference in food preference between diets was observed (Wilcoxon signed-rank test, *p* = 0.001).

**Fig 4:**
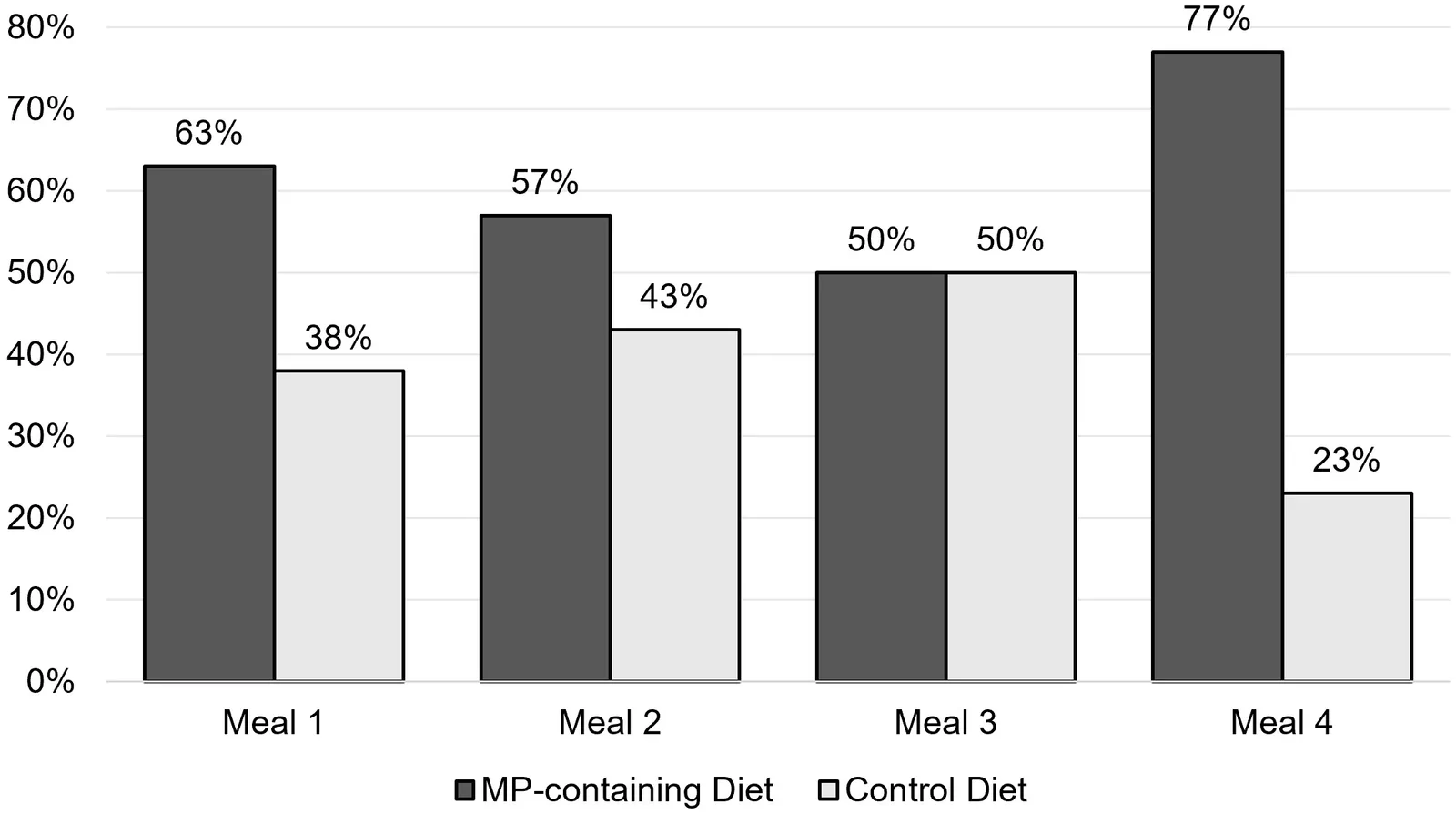
First approach (Cats). Percentage of observations in which cats first approached the MP-containing diet or the control diet during each meal of the two-bowl preference test. Data are presented descriptively as percentages.

**Fig 5:**
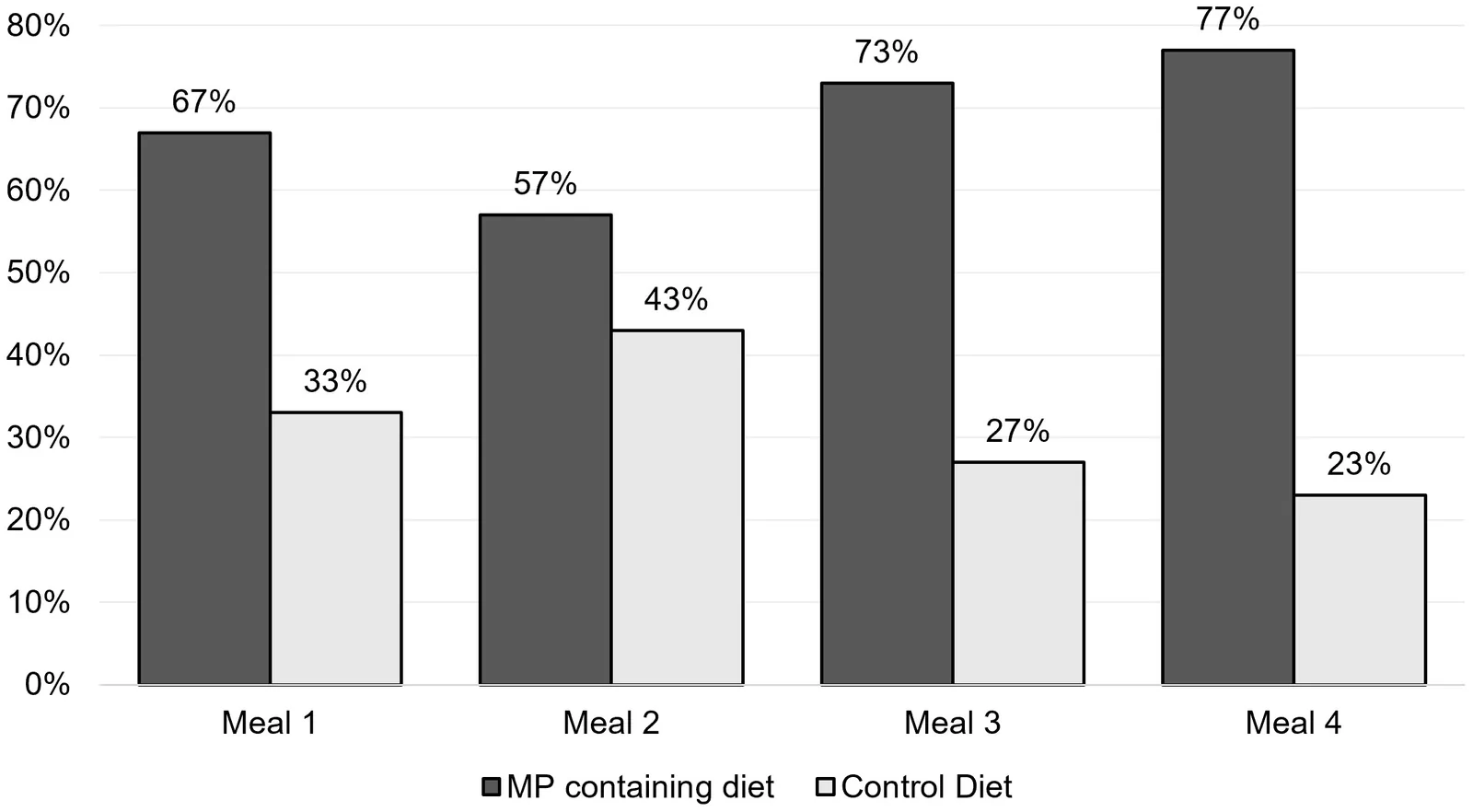
Primary consumption (Cats). Percentage of observations in which cats first consumed the MP-containing diet or the control diet during each meal of the two-bowl preference test. Data are presented descriptively as percentages.

**Fig 6:**
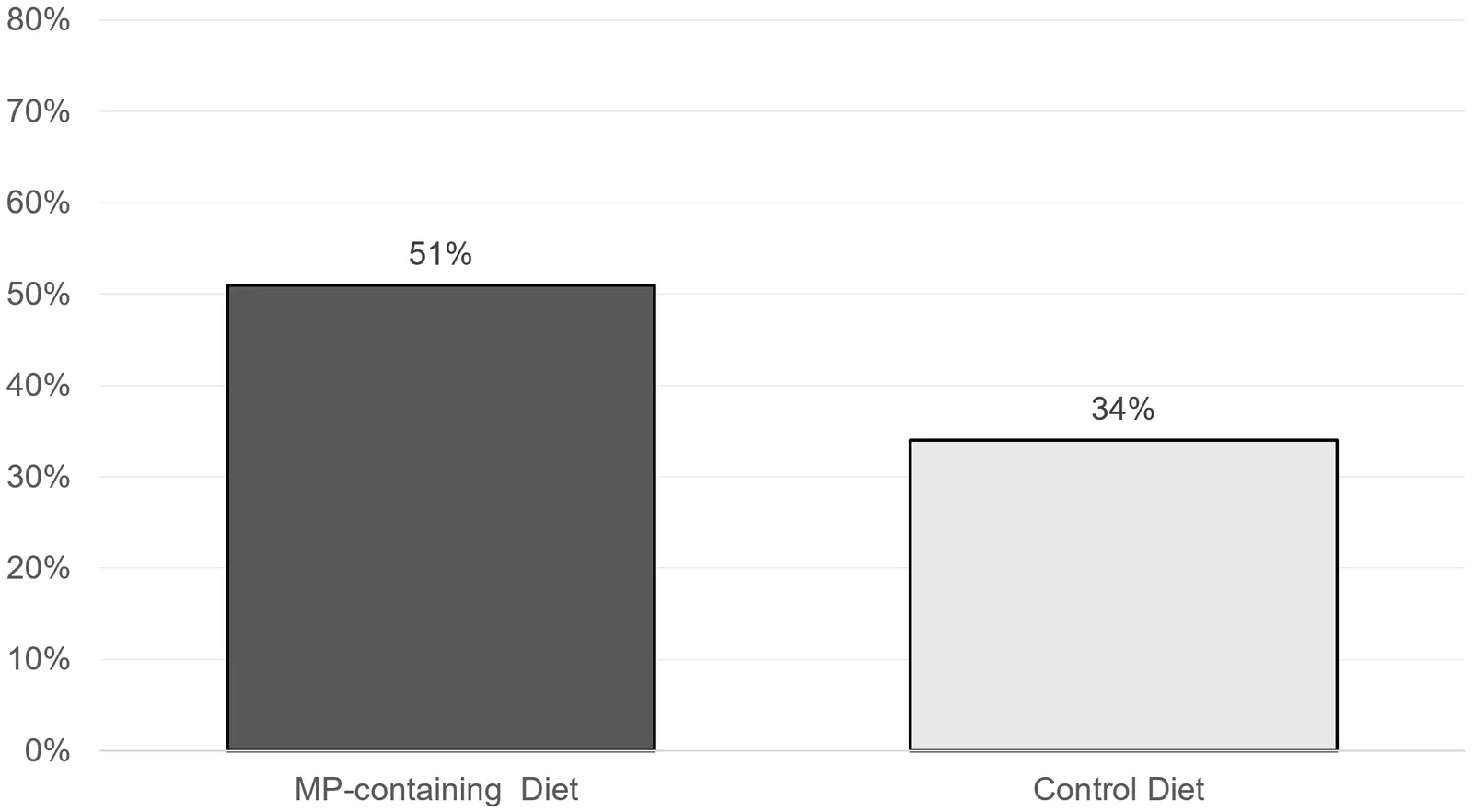
Food Intake (Cats). Mean proportion of the MP-containing diet and control diet consumed by cats during the two-bowl preference test. Bars represent the average percentage of total food intake across the study. Food intake was compared using the Wilcoxon signed-rank test (*p* = 0.001).

On average, across the four meals offered, 62% of cats first oriented toward the bowl containing the MP-containing diet (Fig 4), and 68% consumed this diet first (Fig 5). Mean intake of the MP-containing diet reached 51% of the total offered food, compared with 34% for the control diet (Wilcoxon signed-rank test, p = 0.001; Fig 6).

### Analytical characterization of control and MP-containing diets

Independent laboratory analyses showed that diets containing microbial protein and the control diets were formulated to achieve comparable nutritional targets rather than identical analytical composition, as shown in Table 3. Minor analytical differences were observed, including ash, tryptophan and the ω6:ω3 ratio, maintaining broadly comparable nutritional profiles. Crude fat was equivalent by analysis (13.1% in both diets), while diets containing microbial protein showed a slightly higher ash content (9.8% vs. 9.0% in the control). Amino acid profiling was broadly comparable between diets. Glutamic acid levels were comparable between diets (3.73 vs. 3.71 g/100 g), whereas tryptophan content was higher in the diets containing microbial protein (0.333 vs. 0.259 g/100 g). Fatty acid profiles were similar overall; however, the omega-6: omega-3 ratio was lower in the diets containing microbial protein (8.3 vs. 11.2 in the control). A targeted volatile compound screen did not identify marked elevations of individual volatile compounds in either diet, with most analytes detected below limits of quantification.

**Table 3:** Selected analytical characteristics of the control and MP-containing diets.

Parameter	Control Diet	MP-Containing Diet
Crude Fat (%)	13.1	13.1
Ash (%)	9.0	9.8
Glutamic Acid (G/100 G)	3.71	3.73
Tryptophan (G/100 G)	0.259	0.333
*Ω*6:*Ω*3 Ratio	11.2	8.3
Targeted Volatile Screen	Mostly <Loq	Mostly <Loq

## Discussion

In this study, short-term food preference for dry extruded diets containing MP was evaluated using a two-bowl preference test conducted under an In-Home Use Testing (IHUT) protocol, in comparison with a nutritionally equivalent, macronutrient-matched control diet formulated to differ primarily in protein source. The two-bowl test is a widely established methodology in pet food palatability research and is recognized for its ability to provide reliable and reproducible measures of dietary preference [4]. Animals may develop a preference for a specific side of their feeding area due to habit, spatial comfort, or other environmental factors, which could skew the results of the palatability test. By alternating bowl placement, researchers minimize the influence of positional bias,supporting interpretation of the observed preference in relation to the sensory attributes of the food itself, such as taste, aroma, or texture rather than external factors like bowl positioning [4]. Most participants of the study reported a total food intake similar or even higher than the amounts normally consumed by their pet (both cats and dogs). The significance of owner-reported observations in these trials is underscored by research indicating that pet owners can accurately identify and interpret their animals’ feeding behaviors, which correlates strongly with palatability levels measured through quantitative food consumption [12]. An important aspect of the experimental design is that microbial protein completely replaced fish meal, an ingredient generally regarded as highly palatable in companion animal diets. Despite this substitution, both dogs and cats showed a significant preference for the MP-containing diet under the conditions evaluated. These findings indicate that replacement of fish meal with microbial protein did not compromise short-term food preference under the conditions evaluated. The results indicate that the study protocol did not substantially alter the animals’ usual feeding routine, supporting the interpretation of the observed food preference under routine home-feeding conditions. The four-meal evaluation period was selected to reduce the potential influence of an initial novelty response while maintaining the short-term nature of the preference assessment. Although repeated exposure may lessen transient neophilic or neophobic responses, the present study was not designed to characterize these behavioral phenomena independently, and the observed preference should therefore be interpreted within the context of a short-term food preference test. This study’s findings indicate that diets containing MP were preferred over the control diet under the conditions evaluated. These findings support further investigation of microbial protein as an alternative protein source for pet food.

## Conclusion

In conclusion, this study indicates that the inclusion of microbial protein at 10% did not impair short-term food preference and was associated with increased preference under the conditions evaluated. Diets containing MP were preferred by both dogs and cats compared with the control diet, providing new evidence of short-term food preference in addition to previously reported nutritional quality and digestibility [6,7]. The inclusion of both canine and feline data represents an important contribution, as palatability studies on alternative protein sources have historically focused primarily on dogs. These findings highlight the potential of MP as a versatile ingredient suitable for multiple companion animal species. Together, these findings support further investigation of MP as an alternative protein source for pet food formulations, alongside its previously reported nutritional quality, consistent availability from controlled production systems [6], and favorable food preference. As this study evaluated only short-term food preference, no conclusions can be drawn regarding long-term feeding behavior, sustained acceptance, or health outcomes. Further research involving longer-term feeding trials across different life stages and breeds is warranted to confirm these findings and expand their applicability in companion animal nutrition.

## Supporting information

S1 Table Amino acid composition of the control and MP-containing diets. (DOCX)

S2 Table Mineral composition of the control and MP-containing diets. (DOCX)

S3 Table Targeted volatile compound screening of the control and MP-containing diets. (DOCX)

S1 Dataset Summary data underlying the in-home use test (IHUT) food preference trials in dogs and cats. (DOCX)
