# Supplementary material for: Microbial Protein as a novel ingredient in pet food: A food preference study in dogs and cats
Source: PLoS One. 2026 Sep 23;21(9):e0356767. doi: 10.1371/journal.pone.0356767 (PMC13600530; doi:10.1371/journal.pone.0356767)
Supplement: Supplementary file 1 [file pone.0356767.s001.docx]

**S1 Table. Complete amino acid composition of the control and MP-containing experimental diets.**

| **Amino acid (g/100 g, as-is basis)** | **Control diet** | **MP-containing diet** |
| --- | --- | --- |
| Alanine | 1.75 ± 0.25 | 1.76 ± 0.25 |
| Arginine | 1.82 ± 0.25 | 1.71 ± 0.24 |
| Aspartic acid | 2.21 ± 0.31 | 2.20 ± 0.31 |
| Cysteine + cystine | 0.644 ± 0.090 | 0.474 ± 0.066 |
| Glutamic acid | 3.71 ± 0.52 | 3.73 ± 0.52 |
| Glycine | 2.51 ± 0.35 | 2.38 ± 0.33 |
| Histidine | 0.532 ± 0.074 | 0.496 ± 0.069 |
| Hydroxyproline | 0.436 ± 0.087 | 0.395 ± 0.079 |
| Isoleucine | 1.03 ± 0.14 | 1.02 ± 0.14 |
| Leucine | 2.10 ± 0.29 | 1.98 ± 0.28 |
| Lysine | 1.30 ± 0.18 | 1.32 ± 0.18 |
| Methionine | 0.468 ± 0.066 | 0.455 ± 0.064 |
| Ornithine | <0.05 (LOQ) | <0.05 (LOQ) |
| Phenylalanine | 1.14 ± 0.16 | 1.07 ± 0.15 |
| Proline | 2.13 ± 0.30 | 1.92 ± 0.27 |
| Serine | 1.69 ± 0.24 | 1.47 ± 0.21 |
| Threonine | 1.09 ± 0.15 | 1.05 ± 0.15 |
| Tryptophan | 0.259 ± 0.026 | 0.333 ± 0.033 |
| Tyrosine | 0.773 ± 0.108 | 0.758 ± 0.106 |
| Valine | 1.55 ± 0.22 | 1.50 ± 0.21 |

**S1 Table:** Values are laboratory results reported as value ± analytical uncertainty. Amino acids were determined by ISO 13903:2005 using ion chromatography with UV detection (IC-UV). Cysteine + cystine and methionine were determined following oxidative hydrolysis. Tryptophan was determined separately according to EU Regulation 152/2009 using liquid chromatography with fluorescence detection (LC-FLD). LOQ, limit of quantification.
