# Supplementary material for: Microbial Protein as a novel ingredient in pet food: A food preference study in dogs and cats
Source: PLoS One. 2026 Sep 23;21(9):e0356767. doi: 10.1371/journal.pone.0356767 (PMC13600530; doi:10.1371/journal.pone.0356767)
Supplement: Supplementary file 2 [file pone.0356767.s002.docx]

**S2 Table. Mineral and trace element composition of the control and MP-containing experimental diets.**

| **Mineral / trace element** | **Control diet** | **MP-containing diet** | **Unit** |
| --- | --- | --- | --- |
| Aluminium (Al) | 240 ± 48 | 270 ± 54 | mg/kg |
| Boron (B) | 2.2 ± 0.5 | 1.8 ± 0.4 | mg/kg |
| Cobalt (Co) | <0.2 | 0.4 ± 0.2 | mg/kg |
| Copper (Cu) | 18 ± 4 | 16 ± 6 | mg/kg |
| Iron (Fe) | 320 ± 64 | 280 ± 56 | mg/kg |
| Lithium (Li) | <2.0 | <2.0 | mg/kg |
| Magnesium (Mg) | 1900 ± 380 | 1900 ± 380 | mg/kg |
| Manganese (Mn) | 74 ± 15 | 56 ± 11 | mg/kg |
| Molybdenum (Mo) | <0.2 | 0.6 ± 0.2 | mg/kg |
| Nickel (Ni) | 0.4 ± 0.1 | 0.4 ± 0.1 | mg/kg |
| Potassium (K) | 5200 ± 1000 | 6500 ± 1300 | mg/kg |
| Selenium (Se) | <0.5 | <0.5 | mg/kg |
| Silicon (Si) | >400 | >400 | mg/kg |
| Vanadium (V) | 0.9 ± 0.4 | 0.7 ± 0.4 | mg/kg |
| Zinc (Zn) | 130 ± 26 | 130 ± 26 | mg/kg |

**S2 Table:** Values are laboratory results reported as value ± analytical uncertainty, unless shown as below or above the analytical reporting limit. Minerals and trace elements were quantified by inductively coupled plasma optical emission spectrometry (ICP-OES) using Eurofins internal method CON-PV 00006 (2023-12). Results are reported on an as-is basis.
