# Supplementary material for: Microbial Protein as a novel ingredient in pet food: A food preference study in dogs and cats
Source: PLoS One. 2026 Sep 23;21(9):e0356767. doi: 10.1371/journal.pone.0356767 (PMC13600530; doi:10.1371/journal.pone.0356767)
Supplement: Supplementary file 3 [file pone.0356767.s003.docx]

**S3 Table. Targeted volatile compound screening of the control and MP-containing experimental diets.**

| **Target volatile compound** | **Control diet (mg/kg)** | **MP-containing diet (mg/kg)** |
| --- | --- | --- |
| (E)-Anethole | <10 | <10 |
| alpha-Asarone | <10 | <10 |
| Alpha-bisabolol | <10 | <10 |
| alpha-Pinene | <10 | <10 |
| alpha-Terpinene | <50 | <50 |
| alpha-Terpineol | <10 | <10 |
| alpha-Thujone | <10 | <10 |
| Benzaldehyde | <10 | <10 |
| beta-Asarone | <10 | <10 |
| beta-Myrcene | <10 | <10 |
| beta-Pinene | <10 | <10 |
| beta-Thujone | <5 | <5 |
| Camphor | <10 | <10 |
| Carvacrol | <10 | <10 |
| Carvone | <10 | <10 |
| Caryophyllene | <10 | <10 |
| Cinnamaldehyde | <10 | <10 |
| Citral | <20 | <20 |
| Citronellal | <10 | <10 |
| Citronellol | <10 | <10 |
| Dodecanol | <10 | <10 |
| Estragole | <10 | <10 |
| Eucalyptol (1,8-cineole) | <10 | <10 |
| Eugenol | <10 | <10 |
| Fenchone | <10 | <10 |
| gamma-Terpinene | <10 | <10 |
| Geranial | <20 | <20 |
| Geraniol | <10 | <10 |
| Geranyl acetate | <10 | <10 |
| Isosafrole peak 1 | <10 | <10 |
| Isosafrole peak 2 | <10 | <10 |
| Limonene | <10 | <10 |
| Linalool | <10 | <10 |
| Menthol | <10 | <10 |
| Methyl salicylate | <10 | <10 |
| Methyleugenol | <10 | <10 |
| Neral | <20 | <20 |
| p-Cymene | <10 | <10 |
| Pulegone | <10 | <10 |
| Safrole | <10 | <10 |
| Thymol | <10 | <10 |

**S3 Table**: Targeted aroma-related volatile compounds were quantified by gas chromatography-mass spectrometry (GC-MS) using a validated Eurofins internal method. Values shown with “<” were below the corresponding limit of quantification. Results are reported on an as-is basis. This targeted screening was not designed to identify all volatile compounds or specific positive aroma drivers.
