# Supplementary material for: Microbial Protein as a novel ingredient in pet food: A food preference study in dogs and cats
Source: PLoS One. 2026 Sep 23;21(9):e0356767. doi: 10.1371/journal.pone.0356767 (PMC13600530; doi:10.1371/journal.pone.0356767)
Supplement: Supplementary file 4 [file pone.0356767.s004.docx]

# S4 Dataset. Summary data underlying the in-home two-bowl preference trials

The dog and cat studies were conducted by Sense Test, Lda., an independent contract research organization (CRO). The CRO reports include study procedures, animal characteristics, aggregated descriptive results, statistical analyses, graphical summaries, structured owner-reported outcomes, and de-identified narrative observations.

The underlying animal-level data used to generate the aggregated results, including individual meal-level food intake and behavioral records, were not included in the CRO reports. Consequently, this Supporting Information reproduces and reorganizes all study data available to the authors without estimation, interpolation, reconstruction, or simulation.

Participant and animal names, as well as residential locations, have been removed. Anonymous study-specific identifiers are used throughout this document.

# S4A. Study design

| **Characteristic** | **Cat trial** | **Dog trial** |
| --- | --- | --- |
| CRO report identifier | MH240001 | MH240002 |
| Study period | 8-18 December 2024 | 8-23 December 2024 |
| Report date | 8 January 2025 | 8 January 2025 |
| Panel size | 30 cats | 30 dogs |
| Setting | Animals' habitual feeding environment (home) | Animals' habitual feeding environment (home) |
| Design | Coded two-bowl preference test | Coded two-bowl preference test |
| Exposure | Four meals | Four meals |
| Test diet | MP-containing diet; CRO code 065 | MP-containing diet; CRO code 623 |
| Control diet | Control diet; CRO code 337 | Control diet; CRO code 289 |
| Product delivery | Coded samples in suitable containers; delivered by CRO personnel or representatives | Coded samples in suitable containers; delivered by CRO personnel or representatives |
| Owner instruction | Conditions and rules explained individually; written instructions supplied | Conditions and rules explained individually; written instructions supplied |
| Consumption recording | Owners recorded consumption of each sample across four meals | Owners recorded consumption of each sample across four meals |

**Table S4A**: Study design and sample identification as reported by the CRO. The MP and control labels used here replace the commercial sample codes to match the terminology of the manuscript.

# S4B-1. Animal population

| **Characteristic** | **Cats** | **Dogs** |
| --- | --- | --- |
| Valid animals, n | 30 | 30 |
| Females, n (%) | 16 (53.3%) | 21 (70.0%) |
| Males, n (%) | 14 (46.7%) | 9 (30.0%) |
| Body weight, mean | 5 kg | 6 kg |
| Body weight, reported range | 2-10 kg | 3-10 kg |
| Birth-year range represented | 2007-2021 | 2008-2021 |
| Animals familiar with product type | Yes | Yes |
| Owner compensation | Owners received an incentive for participation | Owners received an incentive for participation |

**Table S4B-1:** Aggregate characteristics of the valid study populations. Percentages were calculated directly from the reported sex counts; all other entries are transcribed from the CRO reports*.*

## S4B-2. Individual animal characteristics: cats

| **Anonymous ID** | **Sex** | **Breed** | **Birth year** | **Body weight (kg)** |
| --- | --- | --- | --- | --- |
| C01 | F | European Shorthair | 2019 | 5.1 |
| C02 | M | European Shorthair | 2015 | 6.5 |
| C03 | M | European Shorthair | 2016 | 4.0 |
| C04 | F | Siamese | 2016 | 4.0 |
| C05 | M | European Shorthair | 2016 | 5.5 |
| C06 | F | European Shorthair | 2016 | 3.0 |
| C07 | F | European Shorthair | 2019 | 3.6 |
| C08 | M | European Shorthair | 2016 | 4.5 |
| C09 | M | European Shorthair | 2011 | 4.5 |
| C10 | F | European Shorthair | 2016 | 3.5 |
| C11 | F | European Shorthair | 2017 | 6.0 |
| C12 | M | European Shorthair | 2015 | 5.5 |
| C13 | M | Bengal | 2016 | 9.0 |
| C14 | F | European Shorthair | 2020 | 5.0 |
| C15 | M | European Shorthair | 2011 | 9.0 |
| C16 | F | European Shorthair | 2016 | 2.8 |
| C17 | F | Scottish Fold | 2020 | 5.0 |
| C18 | M | European Shorthair | 2007 | 5.0 |
| C19 | F | European Shorthair | 2020 | 6.0 |
| C20 | F | European Shorthair | 2014 | 8.0 |
| C21 | M | Norwegian Forest Cat | 2008 | 10.0 |
| C22 | F | European Shorthair | 2009 | 1.8 |
| C23 | F | European Shorthair | 2013 | 5.0 |
| C24 | F | European Shorthair | 2018 | 10.0 |
| C25 | F | European Shorthair | 2015 | 3.7 |
| C26 | M | European Shorthair | 2016 | 6.0 |
| C27 | M | Scottish Straight | 2013 | 4.5 |
| C28 | M | European Shorthair | 2021 | 3.0 |
| C29 | F | European Shorthair | 2016 | 3.2 |
| C30 | M | European Shorthair | 2009 | 5.0 |

**Table S4B-2:** De-identified characteristics of the 30 cats included in the analysis. Breed terminology was translated and standardized only for readability; no values were added or inferred. Reproductive status and body condition score were not included in the CRO report supplied to the authors.

## S4B-3. Individual animal characteristics: dogs

| **Anonymous ID** | **Sex** | **Breed** | **Birth year** | **Body weight (kg)** |
| --- | --- | --- | --- | --- |
| D01 | F | Shih Tzu | 2019 | 5.0 |
| D02 | F | Yorkshire Terrier | 2012 | 4.0 |
| D03 | M | Portuguese Podengo | 2015 | 7.2 |
| D04 | F | Miniature Pinscher | 2019 | 4.5 |
| D05 | F | Mixed breed | 2013 | 7.9 |
| D06 | F | Mixed breed | 2014 | 8.0 |
| D07 | M | Yorkshire Terrier | 2014 | 4.0 |
| D08 | M | Yorkshire Terrier | 2015 | 6.8 |
| D09 | F | Miniature Pinscher | 2018 | 3.0 |
| D10 | M | Poodle | 2014 | 8.0 |
| D11 | F | Doberman Pinscher | 2019 | 2.5 |
| D12 | F | Chihuahua | 2015 | 3.0 |
| D13 | F | Yorkshire Terrier | 2015 | 4.0 |
| D14 | F | Mixed breed | 2015 | 6.0 |
| D15 | F | Dachshund | 2018 | 9.5 |
| D16 | F | Mixed breed | 2014 | 10.0 |
| D17 | F | Mixed breed | 2020 | 3.0 |
| D18 | M | Mixed breed | 2008 | 8.0 |
| D19 | F | Mixed breed | 2019 | 8.0 |
| D20 | F | Mixed breed | 2019 | 8.1 |
| D21 | M | Mixed breed | 2010 | 4.0 |
| D22 | M | Yorkshire Terrier | 2018 | 3.0 |
| D23 | F | Pekingese | 2021 | 3.6 |
| D24 | F | Mixed breed | 2016 | 10.0 |
| D25 | M | Miniature Pinscher | 2019 | 8.0 |
| D26 | F | Portuguese Podengo | 2020 | 4.8 |
| D27 | F | Yorkshire Terrier | 2018 | 2.85 |
| D28 | F | Poodle | 2018 | 5.0 |
| D29 | F | Mixed breed | 2018 | 8.0 |
| D30 | M | Mixed breed | 2016 | 8.0 |

**Table S4B-3:** De-identified characteristics of the 30 dogs included in the analysis. “Mixed breed” corresponds to the source designation SRD. Reproductive status and body condition score were not included in the CRO report supplied to the authors.

# S4C. Diet consumption outcomes

| **Species** | **Diet** | **Median consumed (g)** | **Interquartile range (g)** | **Mean consumed (g)** | **Mean proportion of offered amount consumed** |
| --- | --- | --- | --- | --- | --- |
| Cats | MP diet | 27.0 | 56.3 | 37.0 | 51% |
| Cats | Control diet | 8.0 | 46.0 | 25.3 | 34% |
| Dogs | MP diet | 48.5 | 68.5 | 66.3 | 72% |
| Dogs | Control diet | 32.5 | 63.2 | 46.8 | 50% |

**Table S4C:** Aggregated food-consumption outcomes across the four meals. Medians, interquartile ranges, means, and proportions are transcribed from the CRO reports. The reports label these as aggregated results and do not provide animal-level or meal-level quantities.

# S4D. Behavioral preference outcomes

| **Species** | **Meal** | **First approach: MP-containing diet** | **First approach: control** | **First consumed: MP -containing diet** | **First consumed: control** |
| --- | --- | --- | --- | --- | --- |
| Cats | 1 | 57% | 43% | 67% | 33% |
| Cats | 2 | 50% | 50% | 57% | 43% |
| Cats | 3 | 77% | 23% | 73% | 27% |
| Cats | 4 | 67% | 33% | 77% | 23% |
| Cats | Global | 63% | 38% | 68% | 32% |
| Dogs | 1 | 40% | 60% | 57% | 43% |
| Dogs | 2 | 70% | 30% | 53% | 47% |
| Dogs | 3 | 60% | 40% | 63% | 37% |
| Dogs | 4 | 63% | 37% | 60% | 40% |
| Dogs | Global | 58% | 42% | 58% | 42% |

**Table S4D:** Descriptive behavioral outcomes by meal and globally. Percentages are reproduced from the CRO graphical summaries. No inferential statistical test or confidence interval for these behavioral proportions was supplied; they should therefore be interpreted descriptively.

**S4E. Owner-reported outcomes**

| **Species** | **Meal** | **Usual amount or more** | **Less than usual** |
| --- | --- | --- | --- |
| Cats | 1 | 73% | 27% |
| Cats | 2 | 77% | 23% |
| Cats | 3 | 77% | 23% |
| Cats | 4 | 73% | 27% |
| Cats | Global | 75% | 25% |
| Dogs | 1 | 93% | 7% |
| Dogs | 2 | 90% | 10% |
| Dogs | 3 | 87% | 13% |
| Dogs | 4 | 93% | 7% |
| Dogs | Global | 91% | 9% |

**Table S4E-1:** Owner assessment of the quantity consumed relative to the animal’s habitual amount. These outcomes are descriptive and were not reported as inferentially tested.

# S4F. Statistical analysis

**Consumption analysis:** Pairwise differences in food consumption between the two diets were evaluated using a non-parametric Wilcoxon signed-rank test. Statistical analyses were conducted using XLSTAT (Lumivero, USA) and Microsoft Excel. The exact p-values obtained were 0.001 for cats and 0.002 for dogs. The underlying animal-level paired dataset, handling of ties or missing observations, and test statistics were not provided to the authors; therefore, independent verification of the statistical analysis is not possible.

**Preference-probability display:** The CRO reports also present a preference probability estimate with a corresponding 95% confidence interval for each diet, together with the interpretation that confidence intervals overlapping 0.5 indicate the absence of a statistically significant preference. However, the numerical probability estimates, and confidence interval limits are not reported and are therefore not reproduced here.

**Behavioral and owner-reported variables:** First approach, first consumed, habitual intake comparison, purchase intention, and stool observations are reported descriptively. No inferential statistical analyses for these variables were provided by the CRO.

# S4G. Data provenance and limitations

| **Item** | **Description** |
| --- | --- |
| Data source | Sense Test, Lda. final reports MH240001 and MH240002, dated 8 January 2025 |
| Available level of detail | Animal-level demographic characteristics; aggregate consumption statistics; meal-specific percentage summaries for behavioral outcomes and habitual-intake assessment; purchase-intention counts; stool observations; and CRO descriptions of methods. |
| Unavailable underlying observations | Individual quantities offered and consumed at each meal; individual first-approach and first-consumed choices by meal; individual habitual-intake responses; and the animal-level paired dataset used by the CRO for the statistical analysis. |
| Privacy protection | Animal names and owner residential locations were removed. Anonymous identifiers preserve one record per animal without revealing personally identifiable information. |
| No reconstruction | No animal-level consumption or preference outcomes were reconstructed from graphical or aggregate data. Aggregate values were transcribed directly from the CRO reports without inferring unavailable observations. |
| Interpretive constraint | The supplementary material documents the information available to the authors and the statistical results reported by the CRO. Because the underlying animal-level paired dataset was not provided, independent verification or reanalysis of the statistical tests is not possible. |

**Table S4G:** Data provenance, availability, and limitations of the supplied CRO dataset.

# Abbreviations

CRO, contract research organization; IHUT, in-home use test; MP, microbial protein.
